# A Multicenter Retrospective Chart Review on the Effectiveness and Tolerability of Electroconvulsive Therapy in Adolescents and Young Adults With Major Depressive Disorder or Bipolar Depression

**DOI:** 10.1097/YCT.0000000000000978

**Published:** 2023-11-15

**Authors:** Nout Schukking, Karel W.F. Scheepstra, Isidoor O. Bergfeld, Jeroen A. van Waarde, Indira Tendolkar, Harm-Pieter Spaans, Annette J. M. Hegeman, Dominique S. Scheepens, Anja Lok

**Affiliations:** From the ∗ Department of Adult Psychiatry, Amsterdam UMC, University of Amsterdam; † Amsterdam Neuroscience, Mood, Anxiety, Psychosis, Stress, and Sleep; ‡ Neuroimmunology Research Group, Netherlands Institute for Neuroscience, Amsterdam; § Department of Psychiatry, Rijnstate Hospital, Arnhem; ∥ Department of Psychiatry, Donders Institute for Brain, Cognition, and Behavior, Nijmegen; ¶ Department of Psychiatry, Parnassia Groep, Den Haag; # Department of Psychiatry, St. Antonius Ziekenhuis, Utrecht, the Netherlands.

**Keywords:** electroconvulsive therapy, adolescent, young adult, major depressive disorder, bipolar depression, effectiveness, AUMC - Amsterdam University Medical Centers, BD - bipolar depression, BT - bifrontotemporal, RUL - right unilateral

## Abstract

**Background:**

Major depressive disorder and bipolar depression in adolescents and young adults are prevalent and major contributors to the global burden of disease, whereas effective interventions are limited. Available evidence is insufficient to assess effectiveness and tolerability of electroconvulsive therapy in depressed adolescents and young adults.

**Methods:**

A retrospective chart review was conducted in patients with major depressive disorder or bipolar depression who underwent electroconvulsive therapy from 2001 to 2021 in 12 centers in the Netherlands. Patients were classified as young (15–25 years) and older adults (26–80 years). Primary outcome was effectiveness, expressed as response (≥50% reduction in rating scale score compared with baseline) and remission. Rating scale scores were cross-sectionally assessed at baseline and at the end of the index course. Outcomes of remitters were included in responders. Secondary outcome was occurrence of subjective cognitive impairment and adverse events. Long-term outcomes were not available.

**Results:**

In the young (n = 57) and older adult (n = 41) group, 40.4% and 56.1% (*P* = 0.153) of patients achieved response and 28.1% and 39.0% (*P* = 0.281) remission, respectively. Subjective cognitive impairment (80.5% vs 56.3%; *P* = 0.001) and transient cardiac arrhythmia (14.6% vs 2.8%; *P* = 0.020) were reported significantly more frequently in the older adult group.

**Conclusions:**

Despite significantly more comorbidity of personality disorders, autism spectrum disorders, and anxiety disorders, effectiveness in the young was similar to the older adults. Tolerability was even superior in the young, despite significantly more bilateral treatment. Electroconvulsive therapy could be considered a viable treatment option in depressed adolescents and young adults.

Psychiatric disorders are major contributors to the health-related disability of adolescents (13–18 years) and young adults (19–25 years).^1,2^ Moreover, major depressive disorder (MDD) is the leading cause of disability in young people.^3^ Major depressive disorder is common, with estimated 1-year prevalence of 5% in adolescents^4^ and 6.1% (including bipolar disorder and dysthymia) in young adults.^2^ Moreover, suicide in young people (15–29 years), which is associated with MDD, is the second leading cause of death in this age group.^5^

Adolescents and young adults are navigating the potentially perilous developmental years of growing out of childhood and into adulthood.^6^ Some critical developmental steps occur during these transitional years, reflecting changing neurobiology, the tasks of separation and individuation, and the influences of preexisting and concurrent mental health issues. Approximately 40% of recurrent mood disorders find their origin in the adolescence, with first mood episodes usually occurring before the age of 18 years.^7^ Untreated or undertreated depression may severely delay or distort this transitional phase, hence the importance of adequate treatment interventions. Unfortunately, in current clinical guidelines,^8–10^ the interventions for adolescents with MDD are limited to cognitive-behavioral therapy, interpersonal psychotherapy, and selective serotonin reuptake inhibitors.^11^ Treatments with tricyclic antidepressants, serotonin-norepinephrine reuptake inhibitors, and monoamine oxidase inhibitors are less effective, and treatment with selective serotonin reuptake inhibitors may be associated with increased suicide risk in adolescents.^12,13^ The only widely accepted and safe treatments for bipolar depression (BD) in adolescents are the combination of olanzapine and fluoxetine^14^ and lurasidone.^15^ Most strikingly, approximately 30%–40% of adolescents with MDD do not respond to the initial treatment.^13^ Therefore, an important question in clinical practice is how to effectively treat adolescents and young adults with severe depression who have failed to respond to multiple medications, because waiting for response to other agents may result in increased morbidity and even suicide.

Electroconvulsive therapy (ECT) is superior to pharmacotherapy in adults with severe or treatment-resistant MDD, with estimated response and remission rates of 74.2% and 52.3% in randomized clinical trials and 63.7% and 30.3% in community settings, respectively.^8^ In BD, response rates and speed of response are higher compared with MDD, but remission rates seem equivalent.^9^ Although the effectiveness of ECT in adults with affective disorders has been assessed by randomized clinical trials, none have been conducted in adolescents or young adults yet. Current international treatment guidelines^10,16,17^ are cautious in their statements on the use of ECT in adolescents with affective disorders. Mainly, these statements are based on the AACAP (American Academy of Child and Adolescent Psychiatry) practice parameter,^18^ which states that ECT is indicated in adolescents with treatment-resistant MDD or mania with severe, persistent, and significantly disabling symptoms that have failed 2 psychotropic trials.^16^

The cautious tone in aforementioned guidelines seems justified because the current evidence in the literature is scarce and lacks methodological robustness. A comprehensive review of the literature on ECT use in young people (≤18 years) concluded that remission or marked improvement was reported in 63% of cases with MDD (n = 40) and in 73% with BD (n = 51).^19^ Furthermore, the authors conclude that adverse events seemed similar in type and frequency to those described in adults. Unfortunately, overall quality of the reports was poor, and controlled studies were absent. A literature review embedded in the aforementioned AACAP practice parameter included 8 retrospective studies to assess effectiveness.^18^ It was concluded that the overall rate of response to ECT among adolescents varied between 50% and 100%, with a higher response rate in patients with affective disorders, especially psychotic depression. A systematic review on ECT in adolescents^20^ included 5 studies on effectiveness in affective disorders, of which only one was not reported in the aforementioned reviews.^21^ However, a subjective rating scale was used to evaluate effectiveness.

An important barrier for the use of ECT in the young may be the fear that ECTs have a long-term effect on the developing brain leading to deleterious cognitive functions. However, findings suggest that adolescents do not experience lasting neurocognitive impairment when pre- and post-ECT outcomes were compared.^22–25^ Cognitive functioning may even increase after ECT, as cognitive symptoms of depression may decrease with effective treatment. Furthermore, ECT-treated adolescents did not significantly differ in their cognitive functions from other psychiatric controls.^26^ Reported short-term adverse effects of ECT in adolescents were minor and similar to adults, and mainly consisted of headache, nausea, muscle pain, and prolonged seizures (most likely due to lower seizure thresholds in younger patients).^27–29^ Studies assessing tolerability of ECT in young patients are sparse, show small sample sizes, and have low methodological quality.

In sum, the current evidence lacks robustness for an adequate assessment of effectiveness and tolerability of ECT in adolescents and young adults with MDD or BD. To add more power to this assessment, we have conducted a retrospective chart review in 12 centers in the Netherlands. We hypothesized in advance that the effectiveness and tolerability of ECT in adolescents and young adults with MDD or BD in our sample would be similar to the older adult patients.

## METHODS

### Setting

This retrospective chart review was coordinated by the Amsterdam University Medical Centre (AUMC). All Dutch ECT centers were invited to provide data on treatments with ECT in adolescents and young adults for depression between 2001 and 2021. When no response was received, centers were contacted by phone. Besides AUMC, 11 other centers agreed to share data for this analysis. The Medical Ethical Review Committee of the AUMC reviewed the study; however, full review was waived due to its retrospective design. Data were extracted from electronic patient charts after informed consent was obtained from individual patients. Data of one center were previously published in a Dutch journal.^30^

### Inclusion Criteria

Patients with a diagnosis of MDD or BD, classified according to *Diagnostic and Statistical Manual of Mental Disorders*, *Fourth Edition*, *Text Revision* or *Fifth Edition*, and treated with ECT were included.^31–33^ Objective outcome scores considered eligible were 17-item Hamilton Rating Scale for Depression (HRSD_17_),^34^ Montgomery-Åsberg Depression Rating Scale (MADRS),^35^ Beck Depression Inventory-II (BDI-II),^36^ and (Quick) Inventory of Depressive Symptomatology Clinician-rated (Q)IDS-C and Self-Rated (Q)IDS-SR.^37,38^ Only patients with measurements at baseline (ie, acquired within a week before start of ECT) and follow-up (ie, collected in the week after discontinuation of ECT) were included for analysis.

### Exclusion Criteria

Patients were excluded when ECT was terminated before effects could be expected (ie, within 6 ECT sessions) or when a neurodegenerative disease was present (ie, Alzheimer disease and other dementias, Parkinson disease, motor neuron disease, prion disease, Huntington disease, spinocerebellar ataxia, spinal muscular atrophy).

### ECT Procedure

In all participating centers, ECT was conducted according to the Dutch ECT guideline.^16^ In this guideline, it is recommended to switch to bilateral electrode placement when unilateral ECT is shown to be ineffective after 6 to 8 treatments. Furthermore, it is also recommended that a patient needs to be treated until remission or a plateau in recovery is achieved, meaning lack of improvement during 4 consecutive bilateral treatments.

### Data Extraction

An electronic case report form was created with the use of Castor EDC to aid secure data collection. At 8 of the 11 centers, a database of current and previous electronic patient record systems and digitalized paper files was present. Three centers lacked a database infrastructure, and therefore patients were selected based on the recall of current staff psychiatrists. Records were filtered based on the eligibility criteria for age and intervention. Subsequently, individual patient records were screened manually by the first author (N.S.) for eligibility.

Patient data for the older adult group were collected in the coordinating center for a retrospective chart review on the speed of response to ECT in adults and elderly with a diagnosis of MDD or BD.^39^

Records were scrutinized for the descriptive variables age, sex, diagnosis (MDD or BD), episode duration, number of failed previous antidepressant trials, presence of special features (ie, psychotic, melancholic or atypical), baseline depression rating scale score, baseline cognitive score, and comorbid psychiatric diagnoses. Treatment variables were setting (inpatient or outpatient), method of electrode placement (ie, left [LUL], right unilateral [RUL], and bifrontotemporal [BT]), pulse width (brief or ultrabrief), number of ECT sessions, and number of prolonged seizures. Outcome variables were follow-up depression rating scale score, follow-up cognitive score, and occurrence of general (ie, headache, muscle ache, nausea/vomiting, dental damage, allergic reaction to used hypnotic or muscle relaxant, aspiration, prolonged apnea, prolonged seizure, arrhythmia, and hypotension or hypertension), neurologic (ie, anterograde or retrograde amnesia, aphasia, apraxia, agnosia, dyskinesia, and nonconvulsive status epilepticus), and psychiatric (ie, disinhibition and postictal delirium or psychosis) adverse events.

Data were divided in 2 groups based on age. Age in the “young” group was 15 to 25 years based on criteria for transitional aged youth^6^ and was 26–80 years in the “older adult” group. When patients had received more than 1 ECT course, only data on the first course were included.

### Outcomes

Primary end point was effectiveness of ECT, expressed as treatment response and remission of depressive symptoms and absolute change scores on depression rating scales. Treatment response was defined as ≥50% reduction of score on the used depression rating scale compared with baseline. Remission after the ECT course was defined as HRSD_17_ score ≤7, MADRS score ≤10, BDI-II score ≤9, IDS-C score ≤11, IDS-SR score ≤13, and QIDS-C or QIDS-SR score ≤5. Eventually, scores of the used depression rating scale were converted to HRSD_17_ scores to calculate means.^37,38,40,41^ Secondary end point was tolerability of ECT, defined as the occurrence of general, neurologic, and psychiatric adverse events and the Mini-Mental State Examination (MMSE) score change after the ECT course.

### Statistical Methods

Statistical analyses were performed using SPSS, version 27 and STATA IC, version 15. To examine differences between group means of continuous variables, Student independent *t* test was used when distribution was parametric and Mann-Whitney *U* test when distribution was nonparametric. To examine differences between groups for dichotomous variables, a Fisher exact test was used. Linear and logistic regression analyses were used to relate continuous and dichotomous outcome variables, respectively, to potential confounders and effect modifiers (listed below). In linear models, the dependent variable was the converted HRSD_17_ change score. Dichotomous dependent variables in logistic models were the achievement of response and remission.

We selected several confounders and effect modifiers to include as predictors in the regression models based on the predictive power for response in adults. Increased episode duration, medication failure in the current episode, and comorbid personality disorder were considered robust predictors of lower response rates.^42,43^ Superior effectiveness was expected in patients with psychotic features, in those with a more severe depression, and in those with older age.^8^ Furthermore, the predictive effect of the number of episodes, the age of onset, sex, and a bipolar diagnosis on the effectiveness of ECT seemed to be nonexistent.^42,44^ Bifrontotemporal electrode placement was considered more effective than RUL electrode placement.^45^ Ultrabrief pulse (ie, 0.25–0.5 milliseconds) width was reported to elicit lesser immediate cognitive adverse effects with less effective clinical outcomes compared with brief pulse width (ie, >0.5 milliseconds).^46^ Unfortunately, the method of electrode placement could not be considered in the multivariable models, because a strong association between higher effectiveness and RUL electrode placement was found in the univariable analyses, which seemed contradictory with the literature.^47^ This was likely to be explained by the fact that BT placement was mainly considered in patients in which unilateral treatment had failed.

The maximum number of variables in the final regression models was 10% of the number of observations in linear models and 10% of the total number of events for logistic models. When the number of relevant confounders and effect modifiers exceeded this maximum, confounding variables were selected based on the percentage change of the regression coefficient in univariate models. Effect modifiers were only included in the final regression models if the interaction terms in univariate models were statistically significant (*P* < 0.05). Because of the multicenter design of the study, mixed model analysis was used to assess the influence of clustering of data. Treatment site was considered the first level, individual patients were considered the second level. However, likelihood ratio tests showed no significant differences in response, remission, or converted HRSD_17_ change between sites. Therefore, no random intercepts or random slopes were added to the linear or logistic models.

## RESULTS

### Participants

**FIGURE 1:**
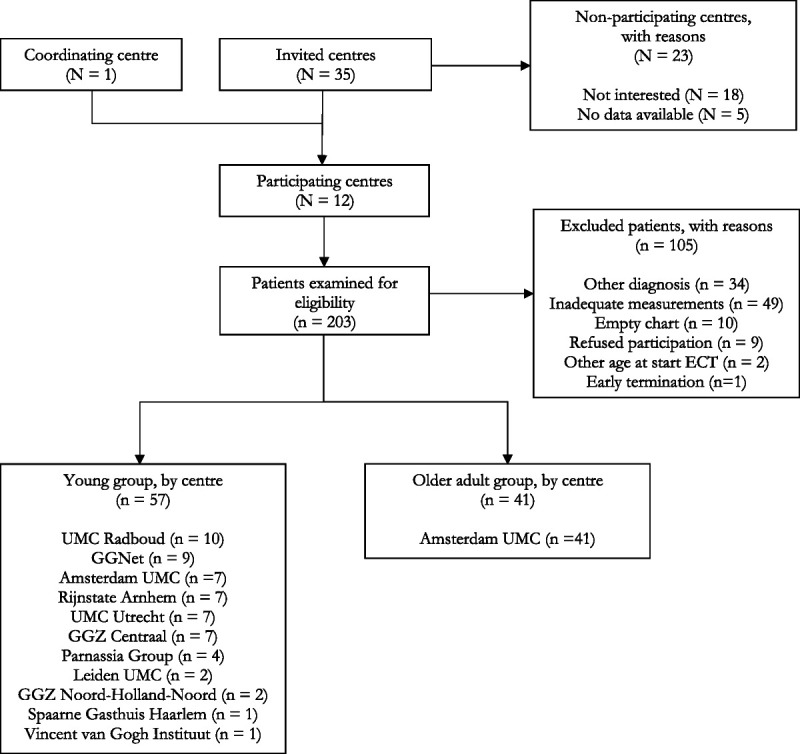
Flow diagram of patient inclusion.

In total, 203 patients were examined for eligibility, of which 98 could be included in the main analyses. Patients were divided in the young (n = 57) and older adult group (n = 41). Reasons for exclusion were diagnosis other than MDD or BD (n = 34), inadequate measurements (n = 49), empty chart (n = 10), refusing participation (n = 9), age outside the range of 15–80 years (n = 2), and early termination of ECT (n = 1). See Figure 1 for a flow diagram of patient inclusion.

### Patient and ECT Characteristics at Baseline

In Table 1, all patient and ECT characteristics of the young and older groups are summarized. In the young group, 21.1% (n = 12) were adolescents, 78.9% (n = 45) were young adults, and the mean ± SD age was 21.0 ± 2.7 years. In the older adult group, 82.9% (n = 34) were adults (age 26–65 years), 17.1% (n = 7) were elderly (age 66–80 years), and the mean ± SD age was 53.9 ± 12.1 years. The young group was treated more often for unipolar depression compared with the older adults (96.5% and 73.2%, respectively; *P* = 0.001). The median depressive episode duration was 13.0 (interquartile range, 6.0–24.0) months in the young, and these data were unavailable in 26.3% of the young and in the complete older adult group. Personality disorders (38.6% vs 7.3%; *P* < 0.001), cluster B personality disorder (19.3% vs 2.4%; *P* = 0.013), anxiety disorders (14.0% vs 0%; *P* = 0.019), autism spectrum disorders (14.0% vs 0%; *P* = 0.019), anorexia nervosa (24.6% vs 0%; *P* ≤ 0.001), and other eating disorders (31.6% vs 0%; *P* < 0.001) were significantly more present in the young group compared with the older adults. The MMSE baseline scores were only available in 35.1% (n = 22) of the young and in 12.2% (n = 5) of the older adult group, and the median score was significantly higher in the young group (30 vs 27; *P* = 0.020).

**TABLE 1:** Patient and ECT Characteristics in Young (15–25 Years) and Older Adults (26–65 Years)

	Young Adults	Older Adults	P
Number, n	57	41	
Adolescents (15–18), % (n)	21.1 (12)	NA	
Young adults (19–25), % (n)	78.9 (45)	NA	
Adults (26–65), % (n)	NA	82.9 (34)	
Elderly (>65), % (n)	NA	17.1 (7)	
Age, mean ± SD (range), y	20.95 ± 2.70 (15–25)	53.9 ± 12.13 (31–79)	<0.001*
Females, % (n)	77.19 (44)	63.4 (26)	0.175†
Unipolar depression, % (n)	96.49 (55)	73.2 (30)	0.001†
Baseline c_HRSD_17_, mean ± SD (range)	25.47 ± 6.99 (8–46)	23.00 ± 6.87 (11–39)	0.085*
Failed previous AD trials, mean ± SD (n)	3.54 ± 1.68 (48)	3.98 ± 2.33	0.311*
Presence of psychotic features, % (n)	21.05 (12)	36.6 (15)	0.111†
Baseline MMSE, median (IQR) (n)	30 (29–30) (20)	27 (20–28) (5)	0.020‡
Episode duration, median (IQR) (n), mo	13.0 (6.0–24.0) (42)	NA	
Personality disorders, % (n)	38.60 (22)	7.3 (3)	<0.001†
Cluster A, % (n)	0.0 (0)	0.0 (0)	
Cluster B, % (n)	19.30 (11)	2.4 (1)	0.013†
Cluster C, % (n)	3.51 (2)	2.4 (1)	0.623†
Unspecified, % (n)	17.54 (10)	4.9 (2)	0.069†
Anxiety disorders, % (n)	14.04 (8)	0.0 (0)	0.019†
General anxiety disorder, % (n)	3.51 (2)	0.0 (0)	0.508†
Social phobia, % (n)	3.51 (2)	0.0 (0)	0.508†
Panic disorder, % (n)	1.75 (1)	0.0 (0)	1.000†
Unspecified, % (n)	7.02 (4)	0.0 (0)	0.137†
Autism spectrum disorders, % (n)	14.04 (8)	0.0 (0)	0.019†
Eating disorders, % (n)	31.58 (18)	0.0 (0)	<0.001†
Anorexia nervosa, % (n)	24.56 (14)	0.0 (0)	<0.001†
Bulimia nervosa, % (n)	0.0 (0)	0.0 (0)	
Unspecified, % (n)	5.26 (3)	0.0 (0)	0.262†
PTSD, % (n)	17.54 (10)	4.9 (2)	0.069†
ADHD, % (n)	3.51 (2)	4.9 (2)	1.000†
OCD, % (n)	1.75 (1)	0.0 (0)	1.000†
SUD, % (n)	3.51 (2)	9.8 (4)	0.233†
Inpatient setting, % (n)	87.72 (50)	58.5 (24)	0.002†
Unilateral electrode placement, % (n)	61.4 (35)	100.0 (41)	<0.001†
Switch to BT, % (n)	38.60 (22)	19.5 (8)	0.049†
Brief pulse width, % (n)	98.25 (56)	NA	
No. treatments, median (IQR)	17.0 (13.0–22.0)	14.0 (10.0–18.0)	0.070‡
Prolonged seizures, % (n)	12.24 (7)	NA	

*Independent *t* test.

†Fisher exact test.

‡Mann-Whitney *U* test.

IQR, interquartile range; NA, not available; AD, antidepressant; PTSD, posttraumatic stress disorder; ADHD, attention-deficit/hyperactivity disorder; OCD, obsessive compulsive disorder; SUD, substance use disorder.

Inpatient treatment was received by 87.7% (n = 50) of the young and 58.5% (n = 24) of older adults (*P* = 0.002). Electrode placement was initially unilateral in 61.4% (n = 35) of the young and 100.0% (n = 41) of older adults (*P* < 0.001). Unilateral electrode placement was switched into BT in 38.6% (n = 22) of the young and 19.5% (n = 8) of older adults (*P* = 0.049). Brief pulse width was used in 98.3% (n = 56) of the young; these data were not collected for the older adults.

### Effectiveness

In Table 2, all outcome parameters are summarized. Effectiveness in the young and older adults did not significantly differ, also after correction for confounding and effect modification. The proportion of patients achieving response was 40.4% in the young and 56.1% in older adults (*P* = 0.153), which corresponds to an odds ratio (OR) of 0.55 (95% confidence interval [CI], 0.24–1.23; *P* = 0.145). After correcting for baseline HRSD_17_ score, the number of failed antidepressant trials, presence of psychotic features, and comorbid personality disorders, the OR appeared 0.72 (standard error, 0.540; 95% CI, 0.251–2.083; *P* = 0.547), which corresponded to adjusted proportions of 49.2% and 57.3% for the young and older adults, respectively. Proportion of patients achieving remission was 28.1% in the young and 39.0% in older adults (*P* = 0.281), which corresponded to an OR of 0.61 (95% CI, 0.26–1.43). Corrected OR for presence of psychotic features and comorbid personality disorders was 0.51 (standard error, 0.263; 95% CI, 0.19–1.40; *P* = 0.193), corresponding to adjusted proportions of 24.9% and 39.5% for the young and older adults, respectively. The mean ± SD change in converted HRSD_17_ score was −10.2 ± 9.3 in the young and −11.1 ± 10.3 in older adults (*P* = 0.668). The mean change in converted HRSD_17_, corrected for converted baseline HRSD_17_ score, number of failed antidepressant trials, presence of psychotic features, and comorbid personality disorders appeared −10.8 and −11.3 for the young and older adults, respectively.

**TABLE 2:** Effectiveness of ECT in Young (15–25 Years) and Older Adults (26–65 Years)

	Young Adults (n = 57)	Older Adults (n = 41)	P
Response, % (n)	40.35 (23)	56.1 (23)	0.153*
c_HRSD_17_, % (n)	42.11 (24)	56.1 (23)	0.220*
HRSD_17_, % (n)	36.59 (15)	56.1 (23)	0.121*
MADRS, % (n)	61.5 (8)	NA	
BDI-II, % (n)	40.0 (4)	NA	
IDS-SR, % (n)	25.0 (1)	NA	
QIDS-SR, % (n)	100.0 (2)	NA	
Remission, % (n)	28.07 (16)	39.0 (16)	0.281*
c_HRSD_17_, % (n)	26.32 (15)	39.0 (16)	0.195*
HRSD_17_, % (n)	26.19 (11)	39.0 (16)	0.247*
MADRS, % (n)	46.2 (6)	NA	
BDI-II, % (n)	18.2 (2)	NA	
IDS-SR, % (n)	0.0 (0)	NA	
QIDS-SR, % (n)	66.7 (2)	NA	
Mean change			
c_HRSD_17_, mean ± SD (n)	−10.19 ± 9.25 (57)	−11.05 ± 10.32 (41)	0.668*
HRSD_17_, mean ± SD (n)	−9.32 ± 8.40 (41)	−11.05 ± 10.32 (41)	0.407*
MADRS, mean ± SD (n)	−17.31 ± 13.30 (13)	NA	
BDI-II, mean ± SD (n)	−11.30 ± 15.86 (10)	NA	
IDS-SR, mean ± SD (n)	−19.00 ± 25.97 (4)	NA	
QIDS-SR, mean ± SD (n)	−14.50 ± 0.71 (2)	NA	

NA, not available; c_HRSD_17_: converted Hamilton Rating Scale for Depression score.

### Tolerability

In Table 3, all included tolerability outcomes are summarized. When comparing general adverse events, cardiac arrhythmia occurred significantly more in the older adult group compared with the young (14.63% vs 2.83%; *P* = 0.020). Dental damage, aspiration, and prolonged apnea did not occur in both groups. Data on general adverse events were unavailable in 15.8% (n = 9) of the young and none of older adults (*P* = 0.009). When comparing neurologic adverse events, subjective cognitive impairment was reported significantly less frequent in the young (56.3% vs 80.49%; *P* = 0.001), and the median follow-up MMSE score was significantly higher in the young compared with older adults (29 vs 27; *P* = 0.019). The MMSE change scores could only be calculated for 26.3% (n = 15) of the young and 2.4% (n = 1) of older adults. In none of the young or older adults, transient neurologic deficits (eg, aphasia, apraxia, agnosia, dyskinesia) occurred. Data on neurologic adverse events were unavailable in 15.8% (n = 9) of the young. Disinhibition and postictal psychosis did not occur in the young or in the older adults. Data on psychiatric adverse events were unknown in 17.54% (n = 10) of the young.

**TABLE 3:** Tolerability of ECT in Young (15–25 Years) and Older Adults (>25 Years)

	Young Adults	Older Adults	*p*
General			
Headache, % (n)	72.92 (35)	53.66 (22)	0.534*
Nausea, % (n)	56.25 (27)	29.27 (12)	0.095*
Muscle ache, % (n)	33.33 (16)	41.46 (17)	0.197*
Allergy to hypnotic, % (n)	4.17 (2)	0.0 (0)	0.508*
Allergy to muscle relaxant, % (n)	2.83 (1)	0.0 (0)	1.000*
Hypotension or hypertension, % (n)	6.25 (3)	7.32 (3)	0.692*
Cardiac arrhythmia, % (n)	2.83 (1)	14.63 (6)	0.020*
Dental damage, % (n)	0.0 (0)	0.0 (0)	
Aspiration, % (n)	0.0 (0)	0.0 (0)	
Prolonged apnea, % (n)	0.0 (0)	0.0 (0)	
Missing, % (n)	15.79 (9)	0.0 (0)	0.009*
Neurologic			
Subjective cognitive impairment % (n)	56.25 (27)	80.49 (33)	0.001*
Transient deficits, % (n)	0.0 (0)	2.44 (1)	0.418*
NSE, % (n)	0.0 (0)	0.0 (0)	
Missing, % (n)	15.79 (9)	0.0 (0)	0.009*
MMSE follow-up, median (IQR) (n)	29 (29–30) (16)	27 (27–29) (5)	0.019†
MMSE change, mean ± SD (n)	−0.07 ± 1.39 (15)	6.0 ± 0 (1)	0.089‡
Psychiatric			
Disinhibition, % (n)	0.0 (0)	0.0 (0)	
Postictal delirium, % (n)	0.0 (0)	4.9 (2)	0.173*
Postictal psychosis, % (n)	0.0 (0)	0.0 (0)	
Missing, % (n)	17.54 (10)	0.0 (0)	0.005*

*Fisher exact test.

†Mann-Whitney *U* test.

‡Independent *t* test.

NA, not available; NSE: nonconvulsive status epilepticus.

## DISCUSSION

### Key Results

In this retrospective chart review, we aimed to establish the effectiveness and tolerability of ECT to treat MDD or BD in adolescents and young adults, and whether these are similar compared with older adults. Although effectiveness seemed higher in older adults (eg, remission rate was 39.0%), no significant difference was shown with the effectiveness in the young patients (eg, remission rate was 28.1%). However, significant differences appeared in the presence of psychiatric comorbidity between groups, as more of the young experienced personality disorders, autism spectrum disorders, anxiety disorders, and eating disorders. Furthermore, the presence of psychotic features was significantly more frequent in the older adults. After correcting for these variables, differences in effectiveness outcomes, especially remission rates, were further reduced. Moreover, despite receiving bilateral treatment more frequently, ECT was better tolerated in the young patients because cardiac arrhythmia and subjective cognitive impairment were reported less frequent. Therefore, our findings suggest that ECT is equally effective in the young as in adults and therefore a useful treatment option in adolescents and young adults with severe depression.

### Interpretation

The response (ie, 40.4%) and remission rates (ie, 28.1%) in our young adult patients were lower compared with most other studies.^22,25,48^ This may be explained by the relatively low presence of psychotic features (ie, in 21.1%) and high occurrence of comorbid personality disorders (ie, in 38.6%) in our young population. In the studies described by Ghaziuddin et al,^18^ Zhand et al,^25^ and Strober et al,^48^ treatment prognostic favorable psychotic features were present in 30.8%, 36.4%, and 100%, respectively, and prognostic unfavorable comorbid personality disorders were present in 16.7% and 42.6%, respectively (not described in Strober et al^48^). The high occurrence of comorbid psychiatric disorders in the young population is likely to be attributed to the low application rate of ECT in the depressed young, because it is only used as treatment-of-last-resort in the most severe or life-threatening cases. Furthermore, it might also be explained by underdiagnosis in the older adult population, which is often the case for autism spectrum disorders for instance.^49^ At last, the prevalence of personality disorders in older adults might be lower because they may have received adequate psychotherapy after which one may not meet the criteria of a personality disorder anymore.^50^

Minor adverse effects (eg, headache, nausea, muscle ache) were common albeit transient in the young as well as in the older adults. The occurrence of prolonged seizures in our young (ie, in 12.2%) corresponded to the findings in other young populations, which is a phenomenon known to occur more frequently in younger than in older adults due to lower seizure threshold in the young patients.^18^ Transient cardiovascular adverse effects (eg, cardiac arrhythmia, hypotension or hypertension) and allergic reactions have been unreported (to the best of our knowledge) in other young populations and seemed sparse and limited in our patients. In our older adults group, and in other older adult patient groups,^51^ transient cardiac arrhythmia occurred more frequently than in the young. This may be explained by the increase of the incidence of arrhythmia with aging. Furthermore, life-threatening arrhythmia might occur in approximately 1.8% of older adults receiving ECT, but did not occur in our study and has not been described in other young patients before.^52^

Occurrence of subjective cognitive impairment in our young patients (ie, in 56.3%) was similar to other young populations^24,25,53^ and was reported significantly less frequently than in our older population (ie, in 80.5%). Interestingly, most (ie, 77.2%) and significantly more of our young patients were (ultimately) treated with BT electrode placement, which is unfavorable for cognitive outcome, compared with the older adults (ie, 19.5%). Bilateral placement might be preferable in patients with a severely endangered clinical condition or severe suicide risk, for a brisker speed of response. Although there are indications that high-dose RUL ECT has a similar antidepressant effect as BT ECT, it cannot be excluded that bilateral placement might be more effective in some patients. Because the mean MMSE change was negligible in the young, these findings support the literature on the absence of objective neurocognitive deficits in young patients shortly after ECT.^22–25^ Although the MMSE is often criticized for having low specificity in detecting cognitive impairment in patients with depression,^54^ it has been widely used in ECT trials and clinical settings.^55^ Because of lack of access to neuropsychological evaluations, we could only present the scores of the MMSE.^56,57^ In a study using more neuropsychological evaluations, ECT-treated adolescents were found to have similar cognitive functions compared with other psychiatric controls.^26^ Future studies should include extensive, standardized pretreatment and posttreatment cognitive assessment, but our current study supports less concern for negative cognitive outcome of ECT in young patients.

### Generalizability

Our findings replicate and extend those of earlier studies that showed effectiveness and tolerability of ECT in the depressed young.^22–24,48,58^ Our study adds data on predominantly young adults to the existing body of evidence on the use of ECT in the depressed young, which until now mainly contained data on adolescents. In our young patients, episode severity was high, treatment resistance was substantial, and often comorbid psychiatric disorders were present. These characteristics are likely to be attributed to the low application rate of ECT in the Netherlands,^59^ which is even lower for the depressed young because it is only used as treatment-of-last-resort in the most severe or life-threatening cases. Therefore, our findings are only applicable to this subgroup of the young. However, because treatment resistance and comorbid personality disorders are inversely associated with effectiveness, we speculate that outcomes may be superior when applied earlier in the treatment course and in the more severely depressed young without psychiatric comorbidity.

### Limitations

Although encouraging, our findings should be considered in the context of the strengths and limitations of the present study, as the used retrospective design had several limitations. Risk for selection bias was present because the uncertainty regarding missing data was introduced and by excluding patients where baseline or follow-up measurements of depression rating scales were unavailable. Furthermore, only baseline and follow-up measurements were used to assess effectiveness, disregarding possible fluctuations of depressive rating scale scores during the treatment course. Because duration and dose of previous antidepressant trials were not always described in detail in the charts, assessment of adequacy of these trials was hindered. Because data of young patients were collected at multiple secondary and tertiary health care sites and data of older adults at a single tertiary health care site, one may argue that this introduces risk for selection bias. However, because ECT in adolescents and young adults is an atypical treatment option, we argue that patient characteristics of this atypical cohort of young patients correspond better to an adult cohort at a tertiary health care site compared with a secondary health care site. At last, sample sizes were too small to achieve sufficient statistical power for noninferiority analysis.

## CONCLUSIONS

Because the burden of depression is high and treatment options are limited for the young, especially in adolescents, ECT needs to be considered as a viable treatment option. When considering this treatment option, clinicians should remain aware of the well-documented, severe adverse outcomes associated with untreated mood disorders in the young.^60–62^ Our data support similar effectiveness of ECT in adolescents and young adults compared with older adults, even in more refractory patients with severe psychiatric comorbidity such as autism spectrum disorders, personality disorders, and anorexia nervosa. Furthermore, our findings do not support severe concerns about negative cognitive effects of ECT in young patients, even when applying BT electrode placement. Hence, we suggest that the application of ECT earlier in the treatment course of depressed adolescents and young adults with severe, persistent, and disabling symptoms deserves more research. To accomplish this, prospective studies with larger sample sizes assessing both short- and long-term effectiveness and cognitive outcomes must be conducted.
